# The Gut-Brain Axis and the Microbiome in Anxiety Disorders, Post-Traumatic Stress Disorder and Obsessive-Compulsive Disorder

**DOI:** 10.2174/1570159X21666230222092029

**Published:** 2023-02-22

**Authors:** Marnie MacKay, Bohan H. Yang, Serdar M. Dursun, Glen B. Baker

**Affiliations:** 1 Department of Psychiatry, Neurochemical Research Unit, University of Alberta, Edmonton, AB, Canada;; 2 Neuroscience and Mental Health Institute, University of Alberta, Edmonton, AB, Canada

**Keywords:** Microbiome, microbiota, gut-brain axis, immune system, prebiotics, probiotics

## Abstract

A large body of research supports the role of stress in several psychiatric disorders in which anxiety is a prominent symptom. Other research has indicated that the gut microbiome-immune system-brain axis is involved in a large number of disorders and that this axis is affected by various stressors. The focus of the current review is on the following stress-related disorders: generalized anxiety disorder, panic disorder, social anxiety disorder, post-traumatic stress disorder and obsessive-compulsive disorder. Descriptions of systems interacting in the gut-brain axis, microbiome-derived molecules and of pro- and prebiotics are given. Preclinical and clinical studies on the relationship of the gut microbiome to the psychiatric disorders mentioned above are reviewed. Many studies support the role of the gut microbiome in the production of symptoms in these disorders and suggest the potential for pro- and prebiotics for their treatment, but there are also contradictory findings and concerns about the limitations of some of the research that has been done. Matters to be considered in future research include longer-term studies with factors such as sex of the subjects, drug use, comorbidity, ethnicity/race, environmental effects, diet, and exercise taken into account; appropriate compositions of pro- and prebiotics; the translatability of studies on animal models to clinical situations; and the effects on the gut microbiome of drugs currently used to treat these disorders. Despite these challenges, this is a very active area of research that holds promise for more effective, precision treatment of these stress-related disorders in the future.

## 1 INTRODUCTION

### 1.1 Stress and Psychiatric Disorders

There is now a large body of evidence indicating that chronic stress has a significant role in the development of several psychiatric disorders, including anxiety disorders and major depressive disorder (MDD) [1-5]. In recent years, there have also been exciting research developments indicating the important role of the gut microbiome and the immune system and their interactions with the brain in the etiology of these psychiatric disorders [6-17] and the effects of various stressors on the composition of the gut microbiome [18-23]. Although there is now extensive research implicating the gut microbiome in a wide variety of diseases and disorders [14], the focus of this review paper is on the interaction of the gut microbiome-immune system-brain axis with anxiety disorders, post-traumatic stress disorder (PTSD) and obsessive-compulsive disorder (OCD). Although all these disorders were listed in the Diagnostic and Statistical Manual of Mental Disorders, version IV (DSM-IV) under Anxiety Disorders, in DSM-5 PTSD and OCD are listed in separate categories. The terms microbiota and microbiome are used interchangeably in this review, although the term gut microbiota refers to the wide variety of microorganisms (bacteria, viruses, fungi and other single-celled organisms) living in the gut, while gut microbiome refers to these microorganisms and their genes. A literature review was done on PubMed and Web of Science for the past 20 years using the search phrases: gut microbiome/ microbiota in: generalized anxiety disorder; panic disorder; social anxiety disorder; post-traumatic stress disorder; obsessive-compulsive disorder. The relevant papers obtained were also searched for other papers related to the gut microbiome-immune system-brain axis.

### 1.2 The Gut Microbiome-immune System-brain Axis

**Fig. (1):**
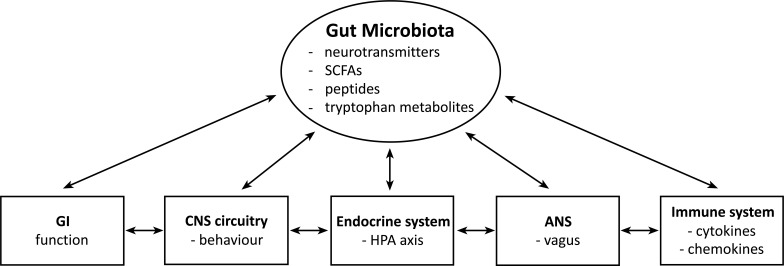
A schematic diagram of the gut microbiome-immune system-brain axis (adapted from Foster and McVey Neufeld [1] with author approval and with copyright clearance from the publisher (Elsevier)). **Abbreviations**: GI = gastrointestinal, CNS = central nervous sytem, HPA = hypothalamic-pituitary-adrenal, ANS = autonomic nervous sytem, SCFAs = short chain fatty acids.

The past couple of decades have seen exciting advances made in learning about the integration of bidirectional communication between the gut microbiome, the immune system and the brain and how that communication is related to psychiatric disorders (Fig. **1**). It has been suggested for some time that the gut microbiome is involved in maintaining the integrity of the mucosal barrier, modulating the immune system and protecting against pathogens [24], and research now provides evidence indicating that alterations in the gut microbiota can modulate plasticity in the brain [1, 25]. It has been reported that the gut microbiome is involved in the regulation of blood-brain barrier (BBB) permeability, brain volume, neurocircuitry, myelination and glial cell function [25, 26]. The gut microbiome can interface with the enteric nervous system and the autonomic nervous system, *e.g*. with the vagus nerve, to send signals to the central nervous system (CNS), and in a bidirectional fashion, the CNS can send signals to the gut to modulate the composition and function of the microbiome [9, 25]. There is constant communication among the mucosal, peripheral and central immune systems and the gut microbiome [8, 27], and the endocrine system also communicates bidirectionally with the gut microbiome, with the hypothalamic-pituitary-adrenal (HPA) axis thought to play a major role [7]. In chronic stress situations, the gut epithelial layer may become more permeable, resulting in increased movement of endotoxins from inside the gut to the outside, leading to a low-grade inflammation characteristic of a number of mood and anxiety disorders [2, 10, 28]. Gut permeability is strongly associated with gut dysbiosis, and increased intestinal permeability to bacterial lipopolysaccharide (LPS) and antigens derived from food can contribute to alterations in functions of glia and the immune system, thus affecting glial regulation of activity and survival of neurons [28]. Comprehensive tables of studies on interactions among the gut microbiome, the immune and endocrine systems and anxiety-like and affective behaviors are included in several recent papers [1, 2, 6, 25].

### 1.3 Microbiome-derived Molecules

Gut microbiota produces a number of active molecules that can affect brain function, although in most cases, the precise mechanisms by which the effects occur are not yet clearly understood. Molecules formed by the gut microbiome and which may alter brain function include neurotransmitters [*e.g*. biogenic amines, acetylcholine and γ-aminobutyric acid (GABA)], short chain fatty acids (SCFAs), indoles (tryptophan and its metabolites), peptides, bile acids, choline and its metabolites, lactate and vitamins [3]. These molecules may affect behavior through various mechanisms, including: direct effects on receptors in the brain; stimulation of neural, endocrine and immune mediators in the periphery; and epigenetic regulation (histone acetylation and DNA methylation) [3, 10, 25]. How the neurotransmitters produced by the gut affect the brain directly [29] is not entirely clear because some do not pass the blood-brain barrier (BBB) readily, but they can modulate the immune system either directly or by affecting cytokine production by local stimulation of the vagus nerve [3, 30-34], possibly affecting brain neurotransmission in this way.

Tryptophan, an essential amino acid that comes from food intake, is the precursor for the neurotransmitter serotonin (5-hydroxytryptamine, 5-HT), which has been proposed to be involved in the etiology and/or treatment of a large number of psychiatric disorders, including major depressive disorder and stress-related disorders that feature symptoms of anxiety [35]. Serotonin is also the precursor of N-acetylserotonin (NAS) and melatonin, which are antioxidants, have antiinflammatory effects and increase oxidative phosphorylation and function in mitochondria [36, 37]. However, much more of the tryptophan in the body is metabolised through the kynurenine pathway. Stress-related increased gut permeability/dysbiosis can cause an increase in levels of proinflammatory cytokines, resulting in activation of indole-2,3-deoxygenase (IDO) and tryptophan-2,3-deoxygenase (TDO), diverting metabolism to the kynurenine pathway, and less tryptophan may be converted to serotonin and subsequently NAS and melatonin. After initial catabolism by IDO, TDO and tryptophanase, tryptophan is converted to numerous metabolites, and several of these indoles can affect neuroinflammation and/or neuro-regeneration and repair [38-45]. Quinolinic acid and kynurenic acid have neurotoxic and neuroprotective properties, respectively [38-42]. Indole, a metabolite of tryptophan produced by gut microbes expressing trpytophanase, has been shown by Wei *et al.* [46] to produce neurogenesis in mouse hippocampuses and to elevate levels of the synaptic markers postsynaptic density protein 95 and synaptophysin. The same researchers reported that indole produces some of these effects through interaction with the aryl hydrocarbon receptor (AhR) and that the indole-AhR-mediated signaling pathway results in elevation of of expression of threee genes involved in neurogenesis, namely, *β-catenin*, *Neurog2* and *VEGF-α*. Kynurenine, in addition to being a precursor for quinolinic and kynurenic acids, has direct effects, including activation of the AhR, thus affecting the function of several immune and glial cells [46].

Anaerobic fermentation of predominantly indigestible carbohydrates by gut microbiota results in the formation of SCFAs, *e.g*. acetate, butyrate and propionate (see Fig. (**2**) for structures). These SCFAs have multiple effects on energy regulation, lipohomeostasis, integrity of the intestinal barrier, the BBB, brain-derived neurotrophic factor (BDNF) expression, functioning of the immune system and epigenetics [3, 25, 47-49]. Wang *et al.* [50] conducted studies in which sodium butyrate was administered orally to control mice and an Alzheimer’s disease (AD) model (the mice were injected with Aβ_25-35_) and found that butyrate improved the cognitive and memory functions of AD mice and also observed that butyrate improved mitochondrial function of astrocytes and enhanced the lactate shuttle between neurons and astrocytes. Stress, by causing increased gut permeability, can result in raised circulating LPS levels and suppressed butyrate levels, driving oxidative and nitrosative signalling, culminating in activation of acidic sphingomyeln-induced ceramide, which has negative effects on the regulation of mitochondrial functions directly and by negatively impacting orexin and pineal gland melatonin, chemicals which normally have a positive regulatory effect on mitochondrial oxidative phosphorylation [51]. Through increasing sirtuin-3 located in the mitochondria, butyrate produces deacetylation and disinhibition of the pyruvate dehydrogenase complex (PDC), resulting in increased conversion of pyruvate to acetyl-CoA, enhancing the production of ATP from the tricarboxylic acid (TCA) cycle and oxidative phosphorylation [51]. Interestingly, acetyl-CoA is also a co-substrate in the initiation of the melatoninergic pathway in the mitochondria, which in turn is instrumental in the regulation of the immune glial cells [52, 53]. O’Riordan *et al.* [48] have provided comprehensive tables listing gut bacteria that produce SCFAs, the effects of SCFAs on brain physiology and the role of SCFAs on enteroendocrine and neuropeptide signalling in the gut-brain axis. Anderson and Maes [51] have published a comprehensive review on how gut dysbiosis may cause dysregulation of central and systemic homeostasis through altering mitochondrial function.

There is also emerging evidence that gut peptides such as neuropeptide Y, peptide YY, antimicrobial peptides, glucagon-like peptide (GLP-1), cholecsytokinin, corticotropin-releasing factor (CRF), oxytocin and ghrelin may play a role in the gut-brain axis [54, 55]. Several of these peptides modulate neuronal and immune functions and also have antimicrobial properties [55]. Gut peptides in systemic circulation can affect gut-brain communication by binding to receptors on immune cells and vagus nerve terminals [54].

An exciting development with regard to microbiome-derived molecules is the discovery that the gut-derived metabolite 4-ethylphenyl sulfate (4-EPS; see Fig. (**2**) for stucture) alters brain activity and functional connectivity in mice and induces anxiety-like activity; the researchers reported that that 4-EPS impairs oligodendrocyte maturation and decreases oligodendrocyte-neuron interactions [56]. A recent study on microbiota-induced alterations in proline metabolism may be relevant to anxiety disorders, although it focuses on depression. Mayneris-Perxachs *et al.* [57], using a multiomics approach, found that microbiome-dependent elevations in plasma levels of proline in humans were associated with the severity of depressive symptoms. In preclinical studies, these researchers found that proline supplementation resulted in increased depression-like symptoms in mice and that knockdown of proline and GABA transporters or mono-association with *L. Plantarum* resulted in protection against depression-like states in *Drosophila*. Although these experiments dealt with depression, the findings may have relevance to anxiety since depression and anxiety are often comorbid and several antidepressants are also anxiolytic; in addition, GABA is known to be important in the etiology and pharmacotherapy of anxiety, and proline accumulation has been shown to disrupt the production of GABA [57].

### 1.4 Probiotics and Prebiotics

Probiotics are consumable microbiota intended for administration to produce a healthier microbiome. Prebiotics include complex carbohydrates and plant polysaccharides that can be catabolized by gut microbiota and can affect several metabolic pathways in the body through the actions of their metabolic by-products, SCFAs [58]. Effects reported with probiotics and prebiotics include dampening of the stress response, decreased intestinal permeability, decreased inflammation, and improvement of the symptoms of depression [5]. Psychobiotics are probiotics or prebiotics that have significant effects on the brain and behavior, particularly on mental health aspects [3, 59-63]. It has been reported by several researchers that probiotics and prebiotics can have antianxiety- and/or antidepressant-like effects in rodent models [1, 33, 63-70] and can produce antianxiety and antidepressant effects, reduced stress hormones, improved cognition and reduced inflammation in humans [71-87]. However, although many of the reported studies in humans are promising, findings are variable and in some cases contradictory, and many papers indicate that larger, better designed clinical trials are necessary [75-77, 82, 84, 87, 88]. High interpersonal variability in microbiome composition also represents a problem for treatment with these preparations, but there are similarities in the relative abundance and distribution of bacterial species among healthy persons [89], giving hope for future treatment of mood and anxiety disorders with microbiota-targeted therapies [6, 90].

## 2 GENERALIZED ANXIETY DISORDER (GAD)

The link between anxiety and the gut microbiome was suggested by studies in mice showing an association between stress reactivity and microbiota [91] and reduced anxiety-like behaviour in germ-free mice lacking microbes [16, 92-94]. Subsequent research on BALB/c mice, which have increased anxiety-like behaviour compared to NIH Swiss mice, showed a reduction in such activity when colonized with microbiota from NIH Swiss mice compared to those colonized with microbiota from donor BALB/c mice; the reverse situation was observed, *i.e*. increased anxiety-like activity, when NIH Swiss mice were colonized with microbiota from BALB/c mice [95]. Interestingly, research in which adult behaviour was studied subsequent to colonization of germ-free mice at various postnatal ages suggested that adolescence might be a key period where microbes can influence the development of stress circuitry and anxiety-like behaviour [1, 16, 92-94, 96]. A study by Desbonnet *et al.* [97] with antibiotics in mice and by Reid *et al.* [98] in fecal analyses from adolescent humans provided additional evidence for the importance of the adolescent period in the development of this anxiety-like behaviour.

Jang *et al.* [99] reported that stress exposure in mice resulted in increased anxiety-like behaviour and increased abundance of *Proteobacteria* and *Escherichia coli* and reduced amounts of *Lactobacillus.* These same researchers demonstrated increased anxiety-like behaviour and inflammation when fecal microbiota was transferred from stressed to unstressed mice, *i.e*. that *E. coli* administered orally resulted in increased anxiety-like activity and that *L. johnsonii* administration reduced anxiety-like behaviour induced by stress or *E. coli.* De Palma *et al.* [100] reported an increased abundance of taxa from the genera *Blautia* to be associated with anxiety in people and mice. They also found that colonization of germ-free mice with fecal microbiota from patients with irritable bowel syndrome (IBS) and accompanying anxiety resulted in increased anxiety-like behaviour in those mice compared to germ-free mice receiving fecal microbiota from healthy human controls or patients with IBS without accompanying anxiety. Jin *et al.* [101] studied anxiety-like behaviour, genetics and gut microbiome composition in the Collaborative Cross (CC) mouse model. They found five microbiota families differing between high and low anxiety mice and proposed that the genetic contribution to anxiety was mediated partially by the gut microbiome [101].

Dandekar *et al.* [102] conducted a comprehensive study in which rats exposed to two models of stress (maternal separation and chronic unpredictable mild stress) were administered the multi-strain probiotic Cognisol for 6 weeks. The probiotic reversed the anxiety as well as a number of biochemical changes produced by the stress, including reversal of decreased brain levels of BDNF and serotonin and restoration of plasma levels of tryptophan and several of its metabolites, the *Firmicutes*-to-*Bacteroide* ratio and fecal levels of SCFAs [102].

Although there is a lack of translational research from animal models to the clinical situation with regard to the gut microbiome and anxiety, altered gut microbial composition, as well as reduced microbial diversity and richness, have been reported in patients with generalized anxiety disorder (GAD) compared to healthy controls [103, 104]. Chen *et al.* [103] found a negative association between the severity of anxiety and abundance of *Prevotella* and a positive association with abundance of *Bacteroides* and *Escherichi-Shigella.* Jiang *et al.* [104] found lower *Prevotellaceae, Faecalibacterium* and *Sutterella* and higher *Lactobacilllus* in GAD patients relative to controls and also reported that GAD patients in remission showed an increase in abundance of SCFA-producing genera (*Eubacterium rectale, Faecalibacterium,* and *Sutterella*) compared to those patients with symptoms of active GAD. Taylor *et al.* [81] reported a positive correlation between *Lactobacillus* and anxiety symptoms. Mason *et al.* [105] found a lower abundance of *Bactriodes* in patients with comorbid anxiety and depression relative to patients with depression only and also reported a negative correlation between *Clostridium XIVa* and anxiety symptoms. In a recent review and meta-analysis, Nikolova *et al.* [106] concluded that there is a transdiagnostic commonality of microbial disturbances in a number of psychiatric disorders, including anxiety, in which there is a depletion of anti-inflammatory butyrate-producing bacteria and an enrichment of proinflammatory bacteria. Guo *et al.* [107] concluded that GAD comorbid with functional gastrointestinal disease (FGID) may be related to an increase in relative abundance of *Fusobacterium* and that GAD not comorbid with FGID may be related to an increase in relative abundance of *Hemophilus*. In a study of 48 patients with GAD and 57 healthy controls, Cheng *et al.* [108] concluded that the onset of GAD is related to BDNF gene polymorphism accompanied by changes in the intestinal flora and in components of the immune system.

Results from several clinical studies on the administration of probiotics and prebiotics to humans have shown improvement in anxiety symptoms and provided further support for a link between gut microbiota and anxiety [109-114], although variable results have been reported. Yang *et al.* [115] conducted a systematic review on the effects of regulating intestinal microbiota on anxiety symptoms. They analyzed 21 studies containing 1,503 subjects; of those studies, 2/3 had probiotics to regulate intestinal microbiota and the others had non-probiotic means such as adjusting daily diets. More than half of the studies found that regulating intestinal flora improved symptoms of anxiety. These researchers also reported that 36% of the studies using probiotics as the intervention were effective, while 86% of the studies in which non-probiotics were used were effective. In their systematic review, Liu *et al.* [75] analyzed 34 controlled clinical trials in which the effects of pre- and probiotics on depression and anxiety were studied. They concluded that prebiotics showed no significant differences from placebo for depression or anxiety, but probiotics showed small significant effects for both depression and anxiety. Chao *et al.* [77], in a meta-analysis of 10 randomized controlled trials, found that probiotics reduced depressive symptoms in patients with anxiety and depression and in healthy people under stress, but there was no reduction in anxiety scores. Noonan *et al.* [76] studied depressive and anxiety symptoms in hemodialysis patients receiving supplementation with a probiotic or synbiotic (combination of pre- and probiotics) for 12 weeks. There was no decrease in anxiety relative to placebo in the probiotic group, but anxiety was reduced in the synbiotic group. Taylor and Holscher [83] carried out a literature review on dietary and microbial connections to depression, anxiety and stress and concluded that fructooligosaccharide and galactooligosaccharide prebiotics in doses ≥ 5 g daily improved anxiety and depression and led to the enrichment of fecal *Bifidobacterium* and that consumption of probiotics improved measures of depression, anxiety or stress in people predisposed to a mood disorder. Smith *et al.* [84] did a systematic review of studies in which participants consumed probiotics, prebiotics or synbiotics and were evaluated for mood or stress levels at the beginning and end of the study. Of the 12 studies investigated, 6 reported reduced depression with probiotics and 2 reported reduced anxiety with probiotics. In a small 8-week clinical study, Eskandarzadeh *et al.* [116] showed a greater decrease in anxiety in patients treated with a combination of a probiotic and the antidepressant sertraline compared to those patients treated with sertraline alone. In a study on 64 healthy females, Johnstone *et al.* [82] reported that administration of a galacto-saccharides prebiotic for 4 weeks increased fecal *Bifidobacterium* abundance and reduced symptoms of anxiety. In a comprehensive review of microbiota imbalance in patients with anxiety and eating disorders and on probiotics, Navarro-Tapia *et al.* [87] concluded that probiotics were more effective in reducing anxiety the higher the baseline anxiety level of the individual. In a small study done by Chinna Meyyappan *et al.* [117] on 12 patients with major depressive disorder (MDD) and/or GAD (8 had MDD and GAD), patients consumed encapsulated MET-2 (contained 40 strains of bacteria purified and lab-grown from the stool of a healthy donor) once daily for 8 weeks; 9 of the patients showed improvement of depression and/or GAD.

## 3 PANIC DISORDER AND SOCIAL ANXIETY DISORDER

Although these two disorders have high prevalence [118], there is only limited specific information available in the literature on their interactions with the gut microbiome. Quagliato *et al.* [119] reported that an elevation of the kynurenine/tryptophan (KYN/tryptophan) ratio in panic disorder (PD) patients is predictive of poor short-term auditory memory. KYN is a major product of tryptophan metabolism and the precursor for several other indolic metabolites, including quinolinic acid, which has neurotoxic effects [120] and kynurenic acid (KYNA), which is a glutamate receptor antagonist [121]. Extensive metabolism of tryptophan is carried out by gut microbiota [122].

Quagliato and Nardi [123], in a systematic review, concluded that serum levels of the proinflammatory cytokines interleukin-6 (IL-6) and IL-1β were increased in PD patients. In a study on 41 PD patients (11 men and 30 women), in which levels of proinflammatory cytokines and cortisol were measured in saliva, it was found that levels of IL-1β, IL-12 and tumor necrosis factor-α (TNF-α) were negatively correlated with cortisol levels [124]; the authors concluded that in PD patients the inflammatory response related to the altered microbiome-gut-brain axis is associated with HPA axis reactivity and may influence the maintenance of anxiety. Although there is a paucity of information on the gut microbiota in PD, alterations in oral microbiota have been reported [125]. There is a large microbiota community in the mouth, and oral microbiome composition has been reported to be associated with adolescent anxiety and depression symptoms [126]. Xie *et al.* [125] carried out 16S rRNA sequencing on the oral microbiota of 26 patients and 40 healthy controls. They found higher alpha diversity (balance and evenness) in PD patients than in healthy controls and differences between the two groups in composition of the oral microbiota. The mean abundances of the genera *Prevotella* and *Veillonella* were observed to be higher in the PD patients than in the healthy controls. It was found that the PD patients and healthy controls differed in the relative abundance of 61 genera. These authors also commented on the reciprocal relationship between PD and the oral microbiota and suggested that PD activates the HPA axis and thereby alters the oral microbial composition.

Butler *et al.* [122] found that the peripheral KYN pathway is altered in social anxiety disorder (SAD), with synthesis being directed preferentially to KYNA synthesis. Relative to healthy controls, SAD patients had elevated plasma levels of KYNA and an increased KYNA/KYN ratio. SAD patients with a history of suicide attempts had increased KYN levels and KYN/tryptophan ratio when compared to those with no such history. These authors found no differences in plasma levels of proinflammatory cytokines between the two groups, but lower levels of the anti-inflammatory cytokine IL-10 were observed in male SAD patients compared to healthy male controls (no such difference was seen between female SAD patients and healthy female controls) [122]. They speculated that the concentration of KYNA in the periphery could be influenced by the gut microbiota, which is known to produce KYNA [127] and inhibit its catabolism [128-130].

## 4 POST-TRAUMATIC STRESS DISORDER (PTSD)

Post-traumatic stress disorder (PTSD), a trauma- and stressor-related disorder, stands alone from other psychiatric disorders as it is the only diagnosis with a known cause -- a direct or indirect exposure to trauma. The exposure can occur either by having a direct experience or witnessing or hearing about repeated traumas and includes actual or threatened death, serious injury or physical violence, sexual violence, or involvement in war, either as a combatant or as a civilian. Motor vehicle accidents and medical traumas are also included, and exposure to natural or man-made disasters is also commonly associated with the development of PTSD. PTSD is characterized by symptom clusters directly associated with the traumatic event. Intrusion symptoms causing distress include memories, vivid dreams and dissociative reactions such as flashbacks, and intense alterations in arousal and physiological reactivity to reminders of the traumatic event are common. Persistent avoidance of situations, places and people linked to the event is also associated with the disorder [131].

Data on the composition of the gut microbiome and microbiota diversity and their relationship to symptoms associated with PTSD are limited. Considering these clinical limitations, the use of a single prolonged stress (SPS) model in rats has been helpful to simulate the behavioural manifestations of PTSD that are present in humans [132] and to examine acute stress-related changes to the microbiome [133]. The model simulates the stress process by exposing the animals to stressors that include bodily restraint, forced swimming and ether exposure [132]. Zhou *et al.* examined the correlation of gut microbiota with behaviour and neurotransmitters in an SPS rat model of PTSD. Behavioural results showed that SPS model rats had significantly increased fear behaviour (measured by mean freezing time) and lower mean times within the parameters measured of the open field test, indicating that anxiety-like behaviour was successfully established under these experimental conditions. Levels of serotonin were significantly decreased, while dopamine (DA) and norepinephrine (NE) levels were increased in the cerebral cortex of the animals [133]. Similar results have been found in the hippocampus and prefrontal cortex (PFC) of PTSD model rats [134]. As serotonin is an inhibitory neurotransmitter, this suggests that the model-exposed animals were in a hyper-excited state which can be synonymous with the arousal symptoms (hypervigilance, high startle response and physiological reactivity) associated with PTSD. Interestingly, there were also differences in the order, family and genus levels in the gut microbiota of SPS-model rats compared to controls. *Firmicutes*, *Bacteroidetes*, *Cyanobacteria* and *Proteobacteria* were the most relevant to the fear- and anxiety-like behaviours and the significant reduction in brain serotonin levels in the cerebral cortex of the SPS model rats [133]. This research demonstrates that PTSD-related behaviours influence the microbiome and impact neurotransmitter concentrations in a validated animal model of PTSD.

In human studies, changes to the gut microbiota and associated PTSD symptoms have been explored in different populations of PTSD-affected individuals. It has been suggested from translational and human research that an acute or chronic stress-induced imbalance in gut microbiota during the neonatal period may have direct effects on the immune and other physiological systems that make those individuals more susceptible to the development of PTSD [135]. Microbial diversity and community composition of the gut microbiome have been investigated in a South African sample of PTSD-affected individuals as compared to trauma-exposed (TE) controls, a particular population where trauma and violence are highly prevalent [136]. Differences were observed, and lower total abundances of the phyla *Actinobacteria*, *Lentisphaerae* and *Verrcumicrobia* [136] were associated with PTSD status.

In more recent times, the experience of the COVID-19 pandemic and the development of PTSD in frontline health care workers (FHWs) by exposure to unprecedented levels of stress has offered a unique opportunity for researchers to study the impact of chronic stress exposure and the gut-brain axis [137]. The influence of prolonged stressors on the gut microbiota and associated PTSD symptoms was investigated in FHWs working on COVID-19 isolation units in Wuhan, China. The mental burden and chronic fear of infection, depression, overwhelming workloads and exposure to unrelenting patient deaths that the FHWs were exposed to were severe. FHWs who worked for two months in specific COVID-19 isolation units were compared to second-line healthcare workers (SHWs) who treated non-COVID-19 infected patients in regular city hospitals. Gut microbiota and mental status after exposure to the COVID-19 frontline work were measured in FHWs immediately after they left the isolation units, after a two-week quarantine period, four weeks after returning home and again at six months after the frontline work. The results indicated that stress associated with frontline work disrupted the gut microbiome at day one as well as produced significant PTSD, anxiety, depression and sleep-related symptoms in FHW, which were more severe than in SHWs. Alpha microbiome diversities (amount of individual bacteria from each bacterial species) were significantly lower in FHWs than in SHWs, indicating that frontline work and the stress associated with it contributed to gut dysbiosis, which persisted during the 6-month follow-up. Fighting against COVID-19 induced long-term changes in gut microbiota underlying the development and recurrence of PTSD symptoms [137].

Symptoms associated with PTSD are often disabling, and to date, there are limited treatment options available. First-line treatments, such as the use of selective serotonin reuptake inhibitors (SSRIs), often fail to improve many of the target symptoms of PTSD. Prazosin, an α_1_-adrenoreceptor antagonist, was a promising candidate, particularly for reducing the occurrence of nightmares and other sleep-related symptoms [138]. Unfortunately, several studies have been unable to replicate these results [139]. D-Cycloserine and propranolol have been specifically used to target the fear circuitry associated with PTSD [140, 141], and synaptic-based strategies such as ketamine administration have also been tried [142]. Psychotherapy has also been used in conjunction with 3,4-methylenedioxymethamphetamine (MDMA, “Ecstasy”) for adult patients with severe PTSD. MDMA has been used successfully in a number of clinical trials and found to be effective and well tolerated. Results have been so impressive that it led the FDA to grant the designation of Breakthrough Therapy for promising treatment in 2017 [143, 144]. A more recent clinical trial studied a flexible dose of MDMA (80 mg or 120 mg) or placebo, followed by a supplemental half-dose (1.5 to 2 hours later with a half-dose of 40 or 60 mg MDMA) administered during 12 weeks of treatment. The treatment period was preceded by 3 preparatory therapy sessions (designed to prepare the patient for the experience) and then the experimental sessions were followed by three integrated sessions of psychotherapy. The effect size of the MDMA-assisted therapy treatment as compared to the placebo/ therapy treatment in this clinical trial was also large at 0.91 and the within-group treatment effect (the effect of the therapy) was 2.1 in the MDMA group and 1.2 in the placebo group respectfully [145]. MDMA, when combined with psychotherapy, may increase tolerance to distressful memories and allow individuals to process the traumatic events without overwhelming symptoms. MDMA increases serotonin release and extracellular serotonin levels by binding to presynaptic serotonin transporters and it has been shown to enhance fear memory extinction and modify fear memory consolidation, retrieval and reconsolidation in mice primarily by an increase in the expression of BDNF in the amygdala [146, 147]. The amygdala involves extensive serotonergic innervation and so it is thought that MDMA’s therapeutic effects may involve modulation of amygdalar serotonin functioning that contributes to fear behaviours and the symptoms of PTSD [145]. MDMA may also impact the gut microbiome. Ridge *et al.* [148] studied the influence of the gut microbiome on the toxicologic effects of hyperthermia in MDMA in rats. Antibiotic treatment for 14 days prior to MDMA treatment was shown to reduce the number and composition of the cecal bacterial population in the animals but also was able to attenuate MDMA-mediated hyperthermia. MDMA also increased expression levels of TGR5 bile acid receptors and enriched the proportion of *Proteus mirabilis* strain in the ceca of those animals not pretreated with antibiotics. The findings suggest that there may be a contributing role of the gut microbiome in MDMA-mediated hyperthermia and that MDMA may also have direct effects on the gut microbiota composition [148].

Unfortunately, physical and medical disabilities such as cardiovascular disease, metabolic syndrome and diabetes mellitus type II are also common in PTSD and require substantial medical and long-term psychiatric support [149]. Other targets focused on inflammatory, metabolic and mitochondrial dysfunctional pathways (all highly associated with the microbiome) have been suggested as these somatic comorbidities are an understudied area of PTSD therapeutics [150]. Drug interventions targeting the microbiome, a key modulator of gut, immune and nervous system functioning, may have a benefit to PTSD-related symptoms, as has been demonstrated in depression and anxiety [116, 151-153]. To date, the use of probiotics has been limited in the treatment of PTSD; however, there is a randomized placebo-controlled clinical trial using *Lactobacillus rhamnosus*, a gram-positive immunoregulatory species that has anti-inflammatory and immunoregulatory properties, that is planned as a probiotic intervention for Veterans with PTSD in the United States (ClinicalTrials.gov Identifier: NCT04150380) [154].

## 5 OBSESSIVE-COMPULSIVE DISORDER (OCD)

### 5.1 The Gut-brain Axis in OCD

The exact causes of OCD still remain unclear, but in recent years research has suggested that immune-mediated mechanisms may be important in at least some cases of OCD [155-159]. In human studies, gut microbiome deviations from normal have been characterized in OCD but also in pediatric autoimmune neuropsychiatric disorders associated with streptococcal infections (PANDAS, a pediatric syndrome in which there is a temporal association between streptococcal infection and acute onset of neuropsychiatric symptoms which include obsessions and compulsions as well as a constellation of cognitive, behavioural, and neurological disturbances [160-162]). In their narrative review on potential immune-microbiome mechanisms in OCD, Troyer *et al.* [155] note that early environmental risk factors for OCD have an overlap with crucial periods related to immune-microbiome development and propose that OCD could be related to the gut microbiome in the following ways: programming of cytokine production, increased trafficking of peripheral immune cells to the CNS and regulation of functioning of microglia. This review also provided comprehensive tables on animal and human studies relating the gut microbiome to OCD-like symptoms; early environmental exposures conferring risk for OCD and also affecting immune-microbiome development; various immune parameters associated with OCD and with changes in the gut microbiota composition in animals or humans; and animal models with potential translation to clinical studies on the immune system and the gut microbiome in OCD [155].

### 5.2 Specific Studies Demonstrating an Altered Microbiome in OCD

A key preclinical study highlighting the differences in microbial composition between obsessive compulsive phenotype deer mice and control type was conducted by Scheepers *et al.* [163]. Specifically, they looked at large nest building as a naturally occurring obsessive compulsive phenotype; this is thought to be a maladaptive behavior of lab mice that confers no survival advantage in the laboratory environment, likely is excessive and expressed at the cost of other functions, and it has been shown to respond to SSRIs [163]. They compared fecal samples between 11 mice with this phenotype to 11 mice without and found the former to demonstrate higher loading of *Desulfovermiculus, Aestuariispira, Peptococcus* and *Holdemanella,* while the latter showed higher loading of *Prevotella* and *Anaeroplasma,* both of which have anti-inflammatory effects [163].

Two clinical studies using RNA sequencing made comparisons between the stool samples of human OCD patients *versus* healthy controls. Turna *et al.* [162] found their OCD group (n = 21) compared to controls (n = 22) had lower alpha diversity and specifically lower relative abundance of three butyrate-producing genera (*Oscillopsira, Odoribacter* and *Anaerostipes*) known to be anti-inflammatory [106, 160]. In addition, they found mean elevations of the inflammatory marker C-reactive protein (CRP) in OCD patients that were associated with the degree of their symptoms [162]. Domenech *et al.* [164] found that in their OCD group (n = 32) compared to controls (n = 32), there was also a similar decrease in alpha diversity [164]. They found increased abundance of the *Rikenellaceae* family associated with MDD and attention deficit hyperactivity disorder (ADHD) as well as gut inflammation [164]. These researchers also found a decrease in the *Coprococcus* genus associated with 3,4- dihydroxyphenylacetic acid (DOPAC) synthesis, a major step in the catabolism of DA. Given that dopaminergic neurotransmission is implicated in OCD, alterations in the metabolism of DA may be a causal link between the decrease in *Coprococcus* and OCD [164].

As further support for these clinical findings, a meta-analysis of 59 case-control studies showed that in adult populations with a range of psychiatric disorders, including MDD, bipolar disorder (BD), schizophrenia, anxiety, OCD, anorexia nervosa, PTSD, and ADHD, there was an overall significant decrease in alpha richness (total number of bacterial species) and a non-significant decrease in alpha diversity [106]. Additionally, they showed that the psychiatric populations had a reduction in anti-inflammatory butyrate-producing bacteria and an increase in pro-inflammatory genera [106].

Furthermore, there has been evidence that suggests acute streptococcal infection leads to the selection of proinflammatory strains of bacteria in children aged 4-8 years old diagnosed with PANDAS. Quagliariello *et al.* [165] found that in their PANDAS group (n = 30) compared to controls (n = 70), the former had significantly decreased bacterial diversity with an increase in abundance of the phylum *Bacteroidetes,* leading to a decreased *Firmicutes* to *Bacteroidetes* ratio [165] which has been associated with intestinal inflammation [166]. It was proposed that this imbalance in gut bacteria ultimately leads to the neuropsychiatric manifestations of PANDAS through the gut-brain axis [165].

It has been postulated by Rees [167] that the use of antibiotics in children with streptococcal infection may lead to the initial gut microbiome imbalance, allowing opportunistic pathogens to thrive, which then creates susceptibility to the development of PANDAS [167]. This theory appears to be in contrast to the theory of PANDAS as a post-infectious phenomenon where antibodies produced through molecular mimicry target the basal ganglia, resulting in OCD symptoms [163, 165, 168]. It also seems to go against what is known in terms of treatment for PANDAS, which involves primarily early and aggressive treatment with antibiotics to control the infection, followed by anti-inflammatory and or psychotropic therapy [169]. Whether the observed gut microbiome imbalance in PANDAS is a direct result of infection or antimicrobials or occurs in the context of a pre-existing condition rendering the individual susceptible to an acute insult warrant further investigation.

### 5.3 Studies that Demonstrate Protective effect of Probiotics on OCD

Given that the above studies suggest a potential causative link between pro-inflammatory gut microbiome profiles and OCD, it stands to reason that perhaps probiotics can have a protective effect by restoring the gut microbial balance. Probiotics have previously been shown to have benefits in inflammatory gastrointestinal (GI) disorders such as inflammatory bowel syndrome (IBS) and diarrhea [166], and thus improving gut inflammation may reduce OCD symptoms *via* the gut-brain axis.

Two significant preclinical studies in rodent models have demonstrated improvement in OCD symptoms with the administration of probiotics. Kantak *et al.* [170] found that oral gavage of house mice with the probiotic bacterium *L. Rhamnosus* at a density of 10^9^ CFU/day for 2 weeks was able to attenuate OCD-like behaviors such as stereotypic movements and marble burying. The OCD-like behaviors were induced with RU 24969 (5HT_1A/1B_ receptor agonist shown previously to exacerbate OCD symptoms in humans) and have been shown to be responsive to serotonin reuptake inhibitors (SRIs) [170]. *L. Rhamnosus* administration was found to be comparable to the SSRI antidepressant fluoxetine at 10 mg/kg for 4 weeks in attenuating OCD-like behaviour [170]. Similarly, Sanikhani *et al.* [171] found that rats with OCD behaviors induced *via* injections with quinpirole (a DA agonist) showed an improvement in OCD signs when fed the probiotic *Lactobacillus casei Shirota* at 10^9^ CF/g/day for 4 weeks, as compared to normal saline [171]. This improvement was also comparable to that of treatment with fluoxetine at 10 mg/kg for 4 weeks [171]. In addition, they found that levels of BDNF (a neurotrophic factor that enhances cellular proliferation, survival rate, and differentiation) in the orbitofrontal cortex (thought to be part of the neurocircuitry implicated in OCD) decreased initially with quinpirole induction of OCD behaviors, but then increased significantly after treatment with either *L. casei Shirota* or fluoxetine [171].

In human studies, Messaoudi *et al.* [112] found that administration of a probiotic formulation consisting of *Lactobacillus helveticus* and *Bifidobacterium longum* for 30 days to a group of 10 healthy human volunteers resulted in decreases in the obsessive-compulsive subscale of the Hopkins symptoms checklist (HSCL-90) as compared to 15 controls [112]. Kobliner *et al.* reported on a case study of a 15-year-old boy with autism spectrum disorder (ASD), OCD, tics, and self-injurious behavior, complicated by a history of GI disturbances and global immune dysfunction. They found that administration of *S. boulardii* up to 72 billion CFU/day resulted in decreased OCD and self-injurious (head-banging) behavior [172]. For further discussion of the studies in OCD mentioned above, readers are referred to a recent review by Marazziti *et al.* [85].

In summary, these studies provide early evidence of the potential benefits of probiotics for OCD symptoms. Despite SSRIs being the primary treatment for OCD, the response rate has been cited as only around 40-60% [170] and these drugs may present with serious side effects including sexual dysfunction [170]. The advent of probiotics, several of which have been reported to support a healthy microbiota, modulate immune responses, and to improve OCD-like behaviours in animal models and humans [112, 170-172], may transform the treatment paradigm of OCD.

### 5.4 Effects of Antidepressants used to Treat OCD on the Gut Microbiome

Interestingly, it appears that the use of common SRIs has also been associated with altered gut microbiomes [173], thereby raising the question of whether they may exert their therapeutic effects for OCD partially through the gut-brain axis. The mechanism has been postulated to be through their broad-spectrum antimicrobial properties, for instance, towards *Staphylococcus, Pseudomonas, Enterococcus*, and *Clostridium* [173]. Sertraline is thought to have the most potent antimicrobial activity; its proposed mechanism is *via* the inhibition of efflux pumps in bacterial cells [173].

Lukic *et al.* [174] conducted a study on BALB/c mice, a strain displaying higher depressive-like behaviour and anxiety relative to other strains. They found that treating mice chronically (21 days) with fluoxetine, escitalopram (SSRIs), venlafaxine, duloxetine (serotonin-NE reuptake inhibitors, SNRIs), or desipramine (a strong NE reuptake inhibitor) resulted in reduced richness (except in the case of desipramine) and increased beta diversity; in particular, they reduced abundances of *Ruminococcus, Adlercreutzia* and an unclassified *Alphaproteobacteria.* [174]. They then supplemented mice treated with duloxetine with *Ruminococcus flavefaciens* and found that this counteracted duloxetine’s diminution of depressive symptoms [174]. The exact mechanism(s) by which *Ruminococcus* leads to more depressive symptoms is unclear, but the treatment of the mice with *R. flavefaciens* upregulated genes involved in mitochondrial oxidative phosphorylation and down-regulated genes involved in neuroplasticity and also led to a decrease of serotonin and NE levels in the medial PFC [174].

## 6 DISCUSSION: CHALLENGES AND FUTURE DIRECTIONS

Although the findings in recent years on the gut-brain axis are exciting and hold great promise for furthering our understanding of the etiology of psychiatric disorders and of improving treatment of these disorders, this is a very complex area of research with many problems still to overcome [88, 115]. When studying the gut microbiome and its interactions with the brain, sex and strain differences in animal models must be taken into consideration [96, 175], as should the possibility that other organs such as the spleen may also have effects on the brain and stress-related disorders by modulating the immune system [176-178]. In humans, further studies must be done on sex-dependent differences in the composition of the gut microbiome and how these relate to anxiety and depression [1, 5, 25, 86]. Malan-Muller *et al.* [13] have provided a comprehensive discussion of potential pitfalls in the design of studies on the gut-brain axis.

Much still remains to be known about how metabolites formed by the gut microbiota that do not cross the BBB affect the brain, although studies on interactions with the vagus nerve are providing important information [20, 34, 107]. Animal models are very useful when studying such problems and trying to tease out other complex interactions among the gut microbiome, the immune system, the autonomic, enteric and endocrine systems and the brain. Animal models are also useful in increasing our knowledge of interactions of the gut microbiome not only with neurons but with glia [50, 179-181]. However, as is often the case with psychiatric disorders, translating from animal models to clinical situations can be problematic. For example, based on animal work, supplementation with SFCAs looks promising for improving anxiety-like and depression-like symptoms, but now more information about translatability to humans, particularly across the lifespan, must be obtained [48].

Great strides have been taken in clinical work on the importance of the gut-brain axis in anxiety disorders, PTSD and OCD, but there are also numerous obstacles to be overcome. These disorders are often comorbid with many other disorders, and future clinical trials must take this into account as well as the sex and drug profile of the patient, length of the trial, diet, exercise and the study populations used [5, 17, 38, 69, 81, 83, 182, 183]. Study populations are important not only from a genetic standpoint but from a bio-psycho-socio-environmental perspective since the gut microbiome may be affected by factors such as lifestyle and increased urbanization; modern urban lifestyles may result in decreased exposure to microbes early in life and result in an underdeveloped immune system and increased risk of inflammation in later life [5, 184], possibly being a risk factor for developing mood and anxiety disorders. Each person’s microbiota profile is unique and may be affected by diet [69, 81, 83, 90, 185]. This uniqueness may in fact, be important in future studies on precision health [90], but we need more specific information about which antianxiety and antidepressant drugs affect particular microbes and the effects of an individual’s genetics on the gut microbiome. Although early studies are promising [186], further information is required to determine if knowledge of the gut microbiome can be used to provide differential diagnoses of disorders like GAD and depression.

Although a great deal of exciting research on the effects of pro- and prebiotics on behaviour has been reported, more knowledge is required on the composition of such preparations that will provide the best therapy for specific disorders [86]. Future studies could use brain imaging methods to examine the effects of various techniques, such as the administration of pro- and prebiotics, used to alter the gut microbiome to see the effects of these techniques on brain function [13, 88, 187]. More information is also required on the influence of alterations of the gut microbiome on specific brain regions such as the amygdala [25]. Although it has been proposed that microRNAs may be potential biomarkers of some psychiatric disorders, there is a necessity to acquire increased knowledge of the effects of exercise and diet on the composition of the gut microbiome and the subsequent effects of excretion of these microRNAs in stools [183]. A major concern that has arisen over the last couple of years has been the long-term effects of COVID-19 on the occurrence of mental health issues; there should be similar concerns on the effects of COVID-19 on the composition of the microbiome in the gut [37, 188]. For example, Yeoh *et al.* [189] obtained blood, stool and patient records from COVID-19 patients and found interesting differences between the patients and non-COVID individuals; the patients had a depletion of gut bacteria with immunomodulatory potential, and the dysbiotic composition of the gut microbiota was still evident at 30 days after clearance of the virus. These researchers also found that the composition of the gut microbiota in the patients was concordant with the severity of the disease and with elevated concentrations of several inflammatory cytokines, chemokines and several blood markers of tissue damage [189]. As mentioned previously (section 4.0) in the current review, frontline workers in COVID-19 isolation units in China have been reported to have gut dysbiosis, which was still evident at a 6-month followup [137]. Fecal microbiota transplantation to treat various conditions, including psychiatric disorders, is an area of great interest, and this topic is discussed in detail in recent review articles [190-192].

In recent years, there has been considerable criticism expressed about the effectiveness of the DSM and the International Classification of Diseases (ICD), which are based on presenting signs and symptoms, in diagnosis of psychiatric disorders because of the heterogeneity of these disorders and the high incidence of co-occurring (co-morbid) disorders. As a result, there is now a strong movement investigating placing more emphasis on neurobiological and behavioural systems in diagnosis and on focusing on personalized health [193-197]. The gut microbiome-brain axis may have an important role in this regard in the future, particularly as technological advances are being made in studying the composition and possible editing of the microbiome [6, 90, 198] and in nutritional neuroscience [199-201]. The findings from such work could also provide physiological explanations for the high incidence of co-morbidities of psychiatric disorders with each other [106] and with other medical conditions [202].

Despite all the challenges mentioned above, research on the gut-brain axis has been a very active and exciting area of endeavor in biological psychiatry over the past two decades and holds promise for increasing our knowledge of the etiology of a variety of stress-related psychiatric disorders, including anxiety disorders, PTSD and OCD, and for providing much-needed biomarkers and improved therapy in the future. Data on the gut-brain axis should become more meaningful as the costs for shotgun metagenomic sequencing and metabolomics continue to fall.

## CONCLUSION

Research on the possible involvement of the gut microbiome-immune system-brain axis in stress-induced disorders such as those described in this review paper is exciting and suggests the possibility of learning more about the etiology of these disorders and providing urgently needed new targets for improved treatment. Many challenges in areas such as comorbidity, improvement of experimental design (*e.g*., with regard to sex, drug profiles, ethnicity/race), environmental effects, the effects of drugs on specific microbes, and translation of findings on animal models to the clinical situation remain, but the findings to date provide strong evidence for directions to take to deal with these challenges in the future.

## Figures and Tables

**Fig. (2):**
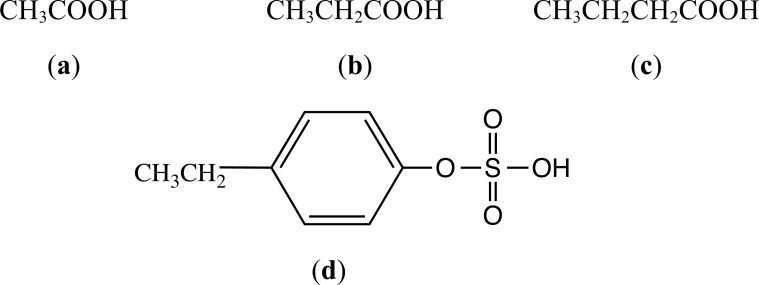
Chemical structures of the short-chain fatty acids (SCFAs) acetic acid (**a**), propionic acid (**b**) and butyric acid (**c**) and of 4-ethylphenyl sulfate (4-EPS) (**d**). The structures were located on a Google Search and drawn using ChemDraw.

## LIST OF ABBREVIATIONS

4-EPS: 4-Ethylphenyl Sulfate
5-HT: 5-Hydroxytryptamine
AD: Alzheimer’s Disease
ADHD: Attention Deficit Hyperactivity Disorder
AhR: Aryl Hydrocarbon Receptor
ASD: Autism Spectrum Disorder
BBB: Blood-brain Barrier
BD: Bipolar Disorder
BDNF: Brain-derived Neurotrophic Factor
CNS: Central Nervous System
CRF: Corticotropin-releasing Factor
CRP: C-reactive Protein
DA: Dopamine
DOPAC: 3,4-Dihydroxyphenylacetic Acid
DSM: Diagnostic and Statistical Manual of Mental Disorders
FGID: Functional Gastrointestinal Disease
FHWs: Frontline Healthcare Workers
GABA: γ-Aminobutyric Acid
GAD: Generalized Anxiety Disorder
GI: Gastrointestinal
GLP-1: Glucagon-like Peptide
HPA: Hypothalamic-pituitary-adrenal
HSCL-90: Hopkins Symptoms Checklist
IBS: Inflammatory Bowel Disease
ICD: International Classification of Diseases
IDO: Indoleamine-2,3-deoxygenase
IL: Interleukin
KYN: Kynurenine
KYNA: Kynurenic Acid
LPS: Lipopolysaccharide
MDD: Major Depressive Disorder
MDMA: 3,4-Methylenedioxymethamphetamine (“Ecstasy”)
NAS: N-acetylserotonin
NE: Norepinephrine
OCD: Obsessive-compulsive Disorder
PANDAS: Pediatric Autoimmune Neuropsychiatric Disorders Associated with Streptococcal Infections
PD: Panic Disorder
PDC: Pyruvate Dehydrogenase Complex
PTSD: Post-traumatic Stress Disorder
SAD: Social Anxiety Disorder
SCFA: Short Chain Fatty Acid
SHWs: Second-line Healthcare Workers
SNRI: Serotonin-norepinephrine Reuptake Inhibitor
SPS: Single Prolonged Stress
SRIs: Serotonin Reuptake Inhibitors
SSRIs: Selective Serotonin Reuptake Inhibitors
TCA: Tricarboxylic Acid
TE: Trauma-exposed
TRP: Tryptophan
